# Editorial: Model-informed drug development and precision dosing in clinical pharmacology practice

**DOI:** 10.3389/fphar.2023.1224980

**Published:** 2023-06-30

**Authors:** Ke Hu, Meng Fu, Xueting Huang, Sumei He, Zheng Jiao, Dongdong Wang

**Affiliations:** ^1^ Jiangsu Key Laboratory of New Drug Research and Clinical Pharmacy and School of Pharmacy, Xuzhou Medical University, Xuzhou, Jiangsu, China; ^2^ Department of Clinical Pharmacology, Jiangsu Hengrui Pharmaceuticals Co., Ltd., Shanghai, China; ^3^ Department of Pharmacy, Suzhou Hospital, Affiliated Hospital of Medical School, Nanjing University, Suzhou, Jiangsu, China; ^4^ Department of Pharmacy, Shanghai Chest Hospital, Shanghai Jiao Tong University School of Medicine, Shanghai, China

**Keywords:** model-informed drug development, model-informed precision dosing, clinical pharmacology, modeling and simulation, practice

Model-informed drug development (MIDD) refers to the integration and quantitative study of physiological, pharmacological, and disease process information using modeling and simulation technology to guide drug development and decision-making, whose aim is to make drug development more efficient and reduce unnecessary patient exposure by integrating data from *in vivo*/*in vitro* studies to predict drug effects (Li et al., 2020). Model-informed precision dosing (MIPD) integrates information related to patients, drugs, and diseases through mathematical modeling and simulation technology to provide a basis for precision medicine for patients. Compared with empirical medication, MIPD is a new method to formulate drug administration schedules based on physiological, pathological, genetic, disease, and other characteristics of patients, which can improve the safety, effectiveness, economy, and adherence of pharmacotherapy (Jiao et al., 2021).

Common models of MIDD and MIPD include but are not limited to the population pharmacokinetics model, pharmacokinetics/pharmacodynamics model, population pharmacokinetics/pharmacodynamics model, physiologically based pharmacokinetics model, quantitative systems pharmacology, model-based meta-analysis, virtual twin, pharmacoeconomic modeling, artificial intelligence, and machine learning.

MIDD and MIPD are essentially the same, given that they solve problems mainly through modeling and simulation. Their differences mainly lie in their different application scenarios, where MIDD mainly refers to modeling and simulation for new drug research and development (Wang et al., 2021; Mitra and Wang, 2022; Chen et al., 2023), while MIPD mainly refers to modeling and simulation for clinical precise drug delivery (Liu et al., 2021; Yin et al., 2022; Li et al., 2023).

Clinical pharmacology is a discipline that studies the law of interaction between drugs and the human body, which based on pharmacology and clinical medicine expounds pharmacokinetics, pharmacodynamics, the nature and mechanism of toxic and side reactions, and the law of drug interaction (Giacomini and Huang, 2022; van der Graaf, 2022; Yao et al., 2022). The main tasks of clinical pharmacology are the clinical research and evaluation of new drugs, reevaluation of market drugs, clinical pharmacokinetic research, adverse drug reaction monitoring, and drug interaction research. With the development of modeling and simulation technology, MIDD and MIPD play an increasingly important role in the practice of clinical pharmacology. Thus, this Research Topic introduced the clinical pharmacological practice of MIDD and MIPD.

Liang et al. found that in critically ill patients, age and albumin level were potentially important factors for the pharmacokinetic parameters of polymyxin B, mainly because older critically ill patients more likely had lower albumin levels, meaning that higher polymyxin B dosage was necessary for efficacy. Cai et al. studied polymyxin B population pharmacokinetics in lung transplantation patients and optimized its administration dosage, finding that renal function had a significant effect on the polymyxin B’s clearance and an adjustment of dosage was needful in lung transplantation patients with renal impairments. Additionally, in the early stage of adult liver transplantation, Cai et al. found the non-linear Michaelis–Menten model could offer credible evidence for tacrolimus dosage optimization in adult liver transplantation patients. Wang et al. reported a joint population pharmacokinetic model of venlafaxine and O-desmethyl venlafaxine in healthy volunteers and patients to estimate the influence of morbidity and drug combination, which may be conducive to achieve precision dosage in clinical pharmacology practice. In Zhu et al.’s research, olanzapine was used as an example to emphasize the feasibility of the real-time estimation of drug concentrations with stacking-based machine learning strategies without losing interpretability, thus further promoting MIPD. Macente et al. determined the dosage regimen recommendation for treatment initiation with sildenafil, specifically in the congenital diaphragmatic hernia indication. Upon treatment initiation, maternal sildenafil dosage should be adjusted on account of therapeutic drug monitoring. Li et al. revealed that toripalimab exposure from a 240 mg Q3W administration dosage was comparable to a 3 mg/kg Q2W administration dosage. In the meantime, the safety and efficacy of 240 mg Q3W was flat, indicating the 240 mg Q3W administration dosage is a preferred therapy dosage for toripalimab based on the convenience of the flat dosage.

In brief, this Research Topic analyzed MIDD and MIPD in clinical pharmacology practice, concentrating principally on modeling and simulation to accelerate drug development and precision dosing.
